# Examining the Use of Text Messages Among Multidisciplinary Care Teams to Reduce Avoidable Hospitalization of Nursing Home Residents with Dementia: Protocol for a Secondary Analysis

**DOI:** 10.2196/50231

**Published:** 2023-08-09

**Authors:** Kimberly R Powell, Mihail Popescu, Suhwon Lee, David R Mehr, Gregory L Alexander

**Affiliations:** 1 Sinclair School of Nursing University of Missouri Columbia, MO United States; 2 School of Medicine University of Missouri Columbia, MO United States; 3 College of Arts and Sciences University of Missouri Columbia, MO United States; 4 School of Nursing Columbia University New York, NY United States

**Keywords:** age-friendly health systems, Alzheimer disease, communication, dementia, nursing homes, older adults

## Abstract

**Background:**

Reducing avoidable nursing home (NH)–to-hospital transfers of residents with Alzheimer disease or a related dementia (ADRD) has become a national priority due to the physical and emotional toll it places on residents and the high costs to Medicare and Medicaid. Technologies supporting the use of clinical text messages (TMs) could improve communication among health care team members and have considerable impact on reducing avoidable NH-to-hospital transfers. Although text messaging is a widely accepted mechanism of communication, clinical models of care using TMs are sparsely reported in the literature, especially in NHs. Protocols for assessing technologies that integrate TMs into care delivery models would be beneficial for end users of these systems. Without evidence to support clinical models of care using TMs, users are left to design their own methods and protocols for their use, which can create wide variability and potentially increase disparities in resident outcomes.

**Objective:**

Our aim is to describe the protocol of a study designed to understand how members of the multidisciplinary team communicate using TMs and how salient and timely communication can be used to avert poor outcomes for NH residents with ADRD, including hospitalization.

**Methods:**

This project is a secondary analysis of data collected from a Centers for Medicare & Medicaid Services (CMS)–funded demonstration project designed to reduce avoidable hospitalizations for long-stay NH residents. We will use two data sources: (1) TMs exchanged among the multidisciplinary team across the 7-year CMS study period (August 2013-September 2020) and (2) an adapted acute care transfer tool completed by advanced practice registered nurses to document retrospective details about NH-to-hospital transfers. The study is guided by an age-friendly model of care called the 4Ms (What Matters, Medications, Mentation, and Mobility) framework. We will use natural language processing, statistical methods, and social network analysis to generate a new ontology and to compare communication patterns found in TMs occurring around the time NH-to-hospital transfer decisions were made about residents with and without ADRD.

**Results:**

After accounting for inclusion and exclusion criteria, we will analyze over 30,000 TMs pertaining to over 3600 NH-to-hospital transfers. Development of the 4M ontology is in progress, and the 3-year project is expected to run until mid-2025.

**Conclusions:**

To our knowledge, this project will be the first to explore the content of TMs exchanged among a multidisciplinary team of care providers as they make decisions about NH-to-hospital resident transfers. Understanding how the presence of evidence-based elements of high-quality care relate to avoidable hospitalizations among NH residents with ADRD will generate knowledge regarding the future scalability of behavioral interventions. Without this knowledge, NHs will continue to rely on ineffective and outdated communication methods that fail to account for evidence-based elements of age-friendly care.

**International Registered Report Identifier (IRRID):**

DERR1-10.2196/50231

## Introduction

### Overview

There are over 15,000 nursing homes (NHs) in the United States, where care is delivered to over 1.3 million residents [1]. Approximately 48% of NH residents have a diagnosis of Alzheimer disease or a related dementia (ADRD) [2]. NH residents with ADRD are at high risk for hospital transfer due to high incidence of comorbidities, polypharmacy, and progressive loss of language, resulting in difficulty communicating preferences [3-5]. Reducing avoidable NH-to-hospital transfers of residents with ADRD has become a national priority due to the physical and emotional toll it places on residents and the high costs to Medicare and Medicaid [6-8]. NH-to-hospital transfers result in expenditures of more than US $2.6 billion annually [9] and in as many as 60% of cases, they are avoidable, meaning the resident could have been safely managed in the NH [10,11]. Early illness recognition and treatment could prevent the need for hospital transfer altogether, ultimately reducing morbidity in residents with ADRD while controlling costs [7,9].

Avoidable NH-to-hospital transfers refer to transfers that follow acute flare-ups of clinical conditions that could have been avoided if appropriate preventative care had been provided in the NH [10]. Recent studies have identified clinical factors such as falls, fever, urinary symptoms, and incontinence that contribute to avoidable transfers [11]. Dementia was the only high-risk clinical condition associated with avoidable NH-to-hospital transfers [11]. Other factors contributing to the decision to transfer, for those deemed avoidable, included a condition that could have been safely managed in the NH, better communication, early detection of new signs or symptoms, and earlier discussion of resident or family preferences [11]. Others have reported communication problems as barriers to timely care for NH residents [12,13]. Specifically, barriers include failure of the communication medium, evening or weekend illness onset with concomitant difficulty contacting an on-call physician, clinical decision-makers who interact through intermediaries, and communication of inappropriate or inaccurate information [13]. Improving communication among health care team members could have a considerable impact on reducing avoidable NH-to-hospital transfers.

NH-to-hospital transfer decision-making is complex and relies on the timely transmission of information among the multidisciplinary team. Unfortunately, many NHs rely on antiquated methods of communication (eg, phone and fax) rather than leveraging modern, convenient, and low-cost options, like exchanging text messages (TMs), which could improve health information sharing, especially in resource-limited settings [12,14-16]. In a pilot analysis using 6 months of TMs exchanged among a multidisciplinary team, the most common messages sent (3244/8946) and received (2319/8946) were about patient updates (updates on symptoms, vital signs, and lab and test results) [17]. However, greater understanding is needed regarding how communication differs between residents with and without ADRD and how TMs can be used among the NH multidisciplinary team to decrease avoidable transfers.

We propose to examine the content of TMs using a framework from Age-Friendly Health Systems that includes 4 evidence-based elements of high-quality care called the 4Ms (What Matters, Medication, Mentation, and Mobility) [18]. Age-Friendly Health Systems is an initiative builds upon a number of fundamental characteristics common to existing geriatric care models, including reliable use of evidence-based care, staff who are specifically trained and proficient in the care of older adults, high-performing care teams focused on measurable outcomes, a systematic approach for coordinating care with other organizations, and engaging patients and their families in a clear process for eliciting goals and priorities and using those goals to individualize care [19]. The 4M framework addresses the core issues that should drive all decision-making in the care of older adults and is a way of systematically rethinking care in ways that improve patient health and satisfaction. A critical need exists to examine how convenient, low-cost communication options like TMs can reduce avoidable NH-to-hospital transfers of residents with ADRD.

### Aims and Research Questions

The objective of this study is to understand how members of the multidisciplinary team communicate and how TMs can be used to avert poor outcomes for NH residents with ADRD, including hospitalization. Our scientific premise is that if multidisciplinary communications are timely and centered around the evidence-based elements of high-quality care for older adults (4Ms), more appropriate decisions will be made, in this case regarding hospital transfer. Thus, the project will consist of complementary steps aimed at (1) identifying documentation of the 4Ms in health information shared by the multidisciplinary team through electronic TMs 2 weeks before NH-to-hospital transfer of residents with ADRD, (2) exploring the association between the 4Ms found in TMs and avoidable NH-to-hospital transfers, and (3) comparing communication patterns of multidisciplinary teams making transfer decisions about residents with and without ADRD.

## Methods

### Overview

Using a retrospective design, we will identify documentation of the 4Ms in health information shared by multidisciplinary teams through electronic TMs and compare communication patterns that occur when making transfer decisions about residents with and without ADRD. This project will use data collected from a Centers for Medicare & Medicaid (CMS)–funded demonstration project designed to reduce avoidable hospitalizations for long-stay NH residents [20,21]. The goals of the project for long-stay NH residents were to reduce the frequency of avoidable hospital admissions and readmissions, improve resident health outcomes, improve the process of transitioning between inpatient hospitals and NHs, and reduce overall health care spending. The project included a multifaceted intervention implemented within 16 NHs in the midwestern United States. The intervention included an advanced practice registered nurse (APRN) embedded full-time within each NH, use of the Interventions to Reduce Acute Care Transfers (INTERACT) tool [22], and an expert support team comprising nurses, social workers, technology experts, and medical directors. Further, the project included enhanced electronic communication using health information exchange, specifically the use of a collaborative text messaging system.

### Ethics Approval

All procedures were approved by the University of Missouri Institutional Review Board (2092039).

### Data Sources

We will use two data sources for this project: (1) TMs and related attributes (eg, sender, recipient, date, and timestamp) and (2) a survey completed by APRNs about NH resident transfers. The TMs were exchanged through a Health Insurance Portability and Accountability Act (HIPAA)–compliant communication and clinical collaboration tool primarily designed to be used among providers, pharmacies, and prescribers. The messaging platform includes the capability to communicate with outside vendors, patients, and anyone seeking to communicate protected health information in a secure environment that meets the requirements of the National Institute of Standards and Technology as well as the Health Information Technology for Economic and Clinical Health Act [23]. Users can access the messaging platform using a browser or mobile device (app). The platform includes workflow features specifically designed for the long-term care and postacute setting, such as read receipts, average response time tracking, adding and removing users from a message, and multiuser accounts with shared names for easy identification. The data set includes sender, recipient, date, and time stamp for each message. Crosswalks have been developed linking individual users to their roles (eg, bedside nurse, unit manager, director of nursing, APRN, and medical doctor [MD]).

The second data source is a Qualtrics survey completed by APRNs that included the INTERACT tool every time a resident transfer occurred during the study period (August 2013-September 2020). Broad categories of the survey included resident characteristics, risk factors for hospitalization, acute change in condition, nonclinical factors contributing to the transfer, and actions taken to evaluate and manage the change in condition before transfer. Individual items within each of the broad categories described common clinical and nonclinical factors that helped clinical staff understand the reasons for the transfer and process improvement considerations to avoid future transfers. APRNs completed the tool for each transfer using a combination of chart review and nurse interviews. These procedures included (1) monthly review of all INTERACT surveys by a team composed of the APRN, project coordinator, and APRN supervisor; (2) identification of resident and NH factors contributing to the transfer; and (3) agreement by the team on the question “Was the transfer potentially preventable (avoidable)?” using the “five whys” quality improvement technique [24]. This technique is an iterative interrogative approach to exploring cause and effect and was used by the team to assure root causes of transfers were considered when establishing agreement on which transfers were deemed avoidable versus unavoidable.

### Sample

We will extract TMs and merge them with resident transfer data for NHs that participated in the CMS demonstration project. Between August 2013 and September 2020, there were 2551 resident transfers, of which 528 had a diagnosis of ADRD. We will extract TMs for 2 weeks before the resident transfer date. This time period was selected because there is evidence that technology can facilitate earlier detection of declining conditions (1-2 weeks earlier) when used to assess older adults [25].

### 4M Ontology Development (Aim 1)

#### Overview

**Figure 1:**
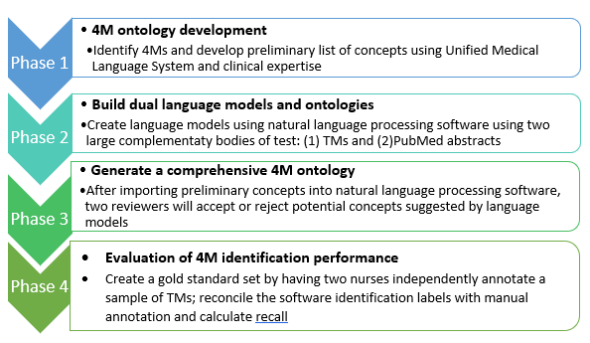
Four phases to generate the 4M ontology. 4M: what matters, medication, mentation, and mobility; TM: text message.

We will use a 4-phased approach (see Figure 1) using standardized vocabularies, clinical expertise, and natural language processing (NLP) to generate a 4M ontology and quantify the 4M content in TMs sent or received 2 weeks before NH resident transfer to the hospital. To enable the extraction of 4M knowledge from TMs, we first need a taxonomy (a set of terms) that formalizes the hierarchical relationships among concepts and specifies the terms to be used to refer to each. From the taxonomy, we can begin to develop an ontology that identifies and distinguishes concepts and their relationships. In information science, ontology is a data model that represents a set of concepts within a domain and the relationships between those concepts [26]. Ontology formalizes the relationships among concepts, which allows computers and humans to interpret the semantic relationships among concepts and infer implicit knowledge [27].

#### Phase 1: 4M Taxonomy Development

While many clinical (eg, Systematized Nomenclature of Medicine Clinical Terms [SNOMED]) and nursing (eg, International Classification for Nursing Practice [ICNP]) standardized languages exist, they might not adequately describe the TMs. The first step in creating our 4M ontology is to use Metamap, the NLP tool provided by the Unified Medical Language System, to extract concepts from our TMs that have relevant semantic types such as “mental process” (menp), “sign or symptom” (sosy), or “clinical drug” (clnd) [28]. The Unified Medical Language System is a compendium of many health terminologies, including SNOMED, INCP, North American Nursing Diagnosis Association, and others, that only extracts concepts with relevant semantic types [29]. Extracted concepts will be reviewed by nurse-clinician scientists with extensive research expertise in NH care and arranged in a taxonomy. The resulting taxonomy will be enriched by the same experts with concepts that might not appear in our TMs. The nurse-clinician scientists will review these lists and make recommendations for changes (eg, addition and removal of concepts and addition and removal of synonyms). The NLP pipeline will be implemented in the Python Natural Language Toolkit and will use the newly developed taxonomy. The NLP pipeline is the set of ordered stages from a labeled data set to create a classifier that can be applied to new samples (ie, supervised machine learning classification). The above procedure will be performed several times, followed by a taxonomy refinement, until at least 90% of the 4M information (ie, 90% recall) is extracted from the entire sample.

#### Phase 2: Building Dual Language Models

We will use two large bodies of text to generate two possibly complementary language models with the NLP software: (1) TMs and (2) PubMed abstracts. These 2 sources were selected because they will allow us to extract a complementary and diverse range of concepts. TMs include clinical jargon and abbreviations, whereas PubMed abstracts have more standardized concepts used in the scientific literature. For the TM source, we will obtain all TMs pertaining to residents with ADRD who had a hospital transfer across the entire CMS demonstration project period (August 2013-September 2020). For the PubMed source, we will extract all available PubMed abstracts containing relevant Medical Subject Headings (MeSH) descriptors. Text from each source will be converted into a single text (.txt) file and uploaded into Metamap. We will use Metamap to perform text preprocessing, convert frequently co-occurring words into triadic expressions (eg, “nurse communicates to physician”), and build language models as described in phase 1.

#### Phase 3: Generating a Preliminary 4M Ontology

We will harmonize the TMs and PubMed versions of the 4M ontology. Two nurse-clinician scientists will independently review and accept or reject suggested concepts for each of the 4Ms. The 2 reviewers will compare lists of words and expressions and discuss discrepancies. If the 2 reviewers cannot come to an agreement on whether a word or expression should be included as a 4M concept synonym, an adjudicator will make the final decision. The output from this step will be a preliminary ontology for each of the 4Ms.

#### Phase 4: Evaluation of NLP 4M Identification Performance Using a Gold Standard Set

We will collect a random sample of TMs, using stratified sampling, from 10% of the resident transfers during the study period. Two nurse-clinician scientists will manually review each TM for the presence or absence of each of the 4Ms. Relative observed agreement (percent agreement between 2 raters) and interrater reliability (Cohen ) will be calculated for each of the 4Ms. The 2 nurses plus a third nurse adjudicator will discuss nonagreement until consensus is achieved. Next, we will use our Natural Language Toolkit NLP software to identify 4Ms in the same gold standard TM set. We will then compare the NLP identification labels to the manual annotation and calculate recall (the NLP software’s ability to identify all notes with a positive occurrence of a particular symptom), precision (proportion of notes with a particular symptom endorsed by NLP), and *F*-measure (a measure of test accuracy that considers both precision and recall). *F*-measures range from 0 (no precision and recall) to 1 (perfect precision and recall). Last, we will review all instances of disagreement between the NLP software identification labels and gold standard annotations. After we validate our 4M extraction pipeline, we will label each TM in our data set with a 4-dimensional vector that would represent the amount of each of the 4Ms that it contains (ie, matter, medication, mentation, mobility). For example, the TM “Resident was depressed after falling and breaking hip” will be labeled as (0,0,1,1). The 4M content will be further used in the analysis for aim 2.

### Exploring the Association Between the 4Ms Found in TMs and Avoidable NH-to-Hospital Transfers (Aim 2)

Using our rich data set, we will compare the representation of the 4Ms in TMs about avoidable and unavoidable transfers while controlling for resident characteristics. We will build a generalized linear model of the Poisson process to compare the representation of the 4Ms in TMs about transfers that were avoidable and unavoidable while controlling for resident characteristics including sex, race or ethnicity, severity of ADRD, and advanced directive status. Our hypothesis is that TMs about avoidable transfers will have more evidence (higher frequency of terms) of the 4Ms compared to TMs about unavoidable transfers*.*

### Comparing Communication Patterns of Multidisciplinary Teams Making Transfer Decisions About Residents With and Without ADRD (Aim 3)

Using the same 2 data sources, we will use social network analysis (SNA) to discern communication and organizational patterns found in TMs occurring in the 2 weeks before NH-to-hospital transfers of residents with and without ADRD. This analysis will allow us to visualize networks and calculate social network measures including density, clustering coefficient, hierarchy, and centralization. Our hypothesis is that TM communication about residents with ADRD will have more decision-making interactions (network density), higher rates of information exchange (clustering coefficient), and more individuals communicating with each other (centralization) compared to TM communication about residents without ADRD. Our premise for this hypothesis is that the gradual loss of language that occurs with ADRD will impact the individual’s ability to communicate with the care team in the earliest stages, regardless of dementia severity [5]. This progressive decline sometimes leaves the person living with ADRD unable to make autonomous decisions. Understanding communication patterns both visually and quantitatively when the multidisciplinary team is making decisions about residents with and without ADRD could help inform decision-making.

## Results

### Data Extraction and Preparation

The initial extraction of TMs resulted in over 77,000 messages that were exchanged by NHs that participated in the CMS demonstration project. We used a stepwise approach to preprocess the TMs. First, we removed TMs where no resident was discussed. For example, there were TMs of a personal nature (eg, “how are you” and “what’s for lunch today”). After removing messages where no resident was discussed, we identified messages about NH residents who had a NH-to-hospital transfer during the study period. NH staff were able to use TMs to communicate about all residents at their facility; therefore, we needed to remove any resident who did not have a hospital transfer during the study period. We excluded over 43,000 messages that were about NH residents that were not in our resident transfer data set, resulting in 30,067 TMs available for analysis. Our next step is to begin identifying the roles of individuals (eg, MD, APRN, bedside nurse, and unit manager) exchanging TMs in preparation for SNA.

### Building the 4M Ontology

**Figure 2:**
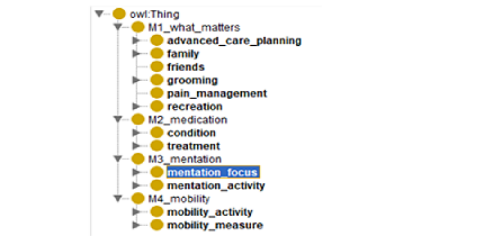
Top level of the 4M ontology. 4M: what matters, medication, mentation, and mobility.

The methodology for building the 4M ontology uses 2 strategies. One strategy is expert-based and top-down, in which the clinical expert outlines the most important concepts for each of the 4Ms. For example, using the concept “what matters,” some concepts we have identified are advanced care planning, family, friends, grooming, pain management, and recreation (see Figure 2). The second strategy is data-driven and bottom-up and consists of extracting meaningful terms from the TMs and inserting them at the right location in the top-level ontology framework created by the expert. The data-driven strategy ensures that we can identify the 4M content of each TM. Expected results to be published in fall 2025.

## Discussion

### Study Implications

To our knowledge, this project will be the first to explore the content of TMs exchanged among a multidisciplinary team of NH care providers as they make decisions about NH-to-hospital resident transfers. Our goal is to apply existing methodologies (SNA and NLP) in a new way, that is, to analyze TMs in NH settings. SNA uses visualization techniques to illustrate patterns of relationships formed among clinical partners, which provides novel quantitative measures of relationship strength and hierarchical structures. NLP is crucial for advancing health care research because it is needed to transform relevant information locked in text into structured data, a prerequisite to quantitative analysis of large amounts of text. The results of the proposed study have the potential to shift both research and clinical paradigms.

This study will produce knowledge of the mechanisms and principles necessary for the successful development and implementation of behavioral interventions using TMs to enhance multidisciplinary team communication in NHs. Knowledge of these principles will make it easier to predict who might respond well to a future intervention and who might not, so that the intervention might be better personalized and given only to those who are likely to benefit from it [30]. Findings from this study, specifically the 4M structured language ontology, could also inform TM interface design, creating the opportunity for standardized, seamless portability of information centered around what matters to older adults. The idea is to address, as much as possible, issues of real-world fidelity and the ability to implement before the intervention is studied at later stages to maximize the chances of success. This approach aligns with the National Institute for Aging Stage Model for Behavioral Intervention Development, with the proposed project falling between stages 0 and 1A [30]. Demonstrating how the 4Ms are represented in TMs must be done before intervention development (stage 0). Understanding how the presence of the 4Ms in TMs relates to avoidable hospitalizations among NH residents with ADRD will inform the evidence base regarding future scalability (stage 1A). Without this knowledge, NHs will continue to rely on ineffective and outdated communication methods that fail to account for evidence-based elements of age-friendly care.

### Limitations

This study will generate knowledge that will have far-reaching implications for NH residents with ADRD. Nonetheless, there are potential limitations to generalizability to consider. Our data set is limited to NHs that participated in the CMS demonstration project. The NHs that participated in the project each had an APRN working as an essential staff member in the facility. Not all NHs have an APRN presence in the facility, so generalizability beyond NHs with this support in place could be limited. To address this limitation, we will consider the wide variability of the use of TMs among APRNs. For example, in our pilot analysis using 6 months of TM data, we found the total number of TM sent by APRNs ranged from 0 to 607 [17]. In the CMS demonstration project, data were captured only for residents who had a NH-to-hospital transfer, so comparing residents who transferred to those who were not transferred is not possible from this data set. To address this limitation, we will compare transfers according to whether they were considered avoidable or unavoidable.

In some cases, NH-to-hospital transfer is appropriate and necessary. Our focus on reducing avoidable NH-to-hospital transfers accounts for this limitation. Although our data set includes novel components, including TMs sent and received between a multidisciplinary care team, resident characteristics, and transfer data, it does not include direct communication with family members of NH residents. To address this limitation, we will account for indirect family communication. For example, family preferences were frequently mentioned in TMs shared among the care team and can be conceptualized with “what matters.” Finally, while the focus of the proposed study is improving communication using TMs, we recognize other factors such as staffing, workload, and skill mix of the NH staff could impact the outcome, that is, avoidable NH-to-hospital transfers. To address this limitation, we will use SNA, which accounts for skill mix by identifying roles (eg, MD, registered nurse, licensed practical nurse, and certified nursing assistant), interactions between social positions and groups, and relationships among groups.

### Conclusions

Identifying evidence-based elements of communication in TMs exchanged among NH health care team members can potentially decrease avoidable hospitalizations and ultimately morbidity and mortality in NH residents with ADRD. The development of a structured language ontology using an evidence-based framework (4M) to improve communication among NH team members is a direct and effective implementation strategy designed to address issues of real-world fidelity before the development of interventions to promote timely and seamless portability of information across the complete spectrum of care, optimizing the health of individuals and populations.

## Appendix

Multimedia Appendix 1 Peer-review report by the Interdisciplinary Clinical Care in Specialty Care Settings Study Section of the University of Missouri-Columbia.

## Abbreviations

4Ms: What Matters, Medication, Mentation, and Mobility
ADRD: Alzheimer disease or a related dementia
APRN: advanced practice registered nurse
CMS: Centers for Medicare & Medicaid
HIPAA: Health Insurance Portability and Accountability Act
ICNP: International Classification for Nursing Practice
INTERACT: Interventions to Reduce Acute Care Transfers
MD: medical doctor
MeSH: Medical Subject Headings
NH: nursing home
NLP: natural language processing
SNA: social network analysis
SNOMED: Systematized Nomenclature of Medicine Clinical Terms
TM: text message

## Data Availability

The data sets generated or analyzed during this study will be available from the corresponding author upon reasonable request.
