# Supplementary material for: Examining the Use of Text Messages Among Multidisciplinary Care Teams to Reduce Avoidable Hospitalization of Nursing Home Residents with Dementia: Protocol for a Secondary Analysis
Source: JMIR Res Protoc. 2023 Aug 9;12:e50231. doi: 10.2196/50231 (PMC10448283; doi:10.2196/50231)
Supplement: Multimedia Appendix 1 [file resprot_v12i1e50231_app1.pdf]

SUMMARY STATEMENT
(Privileged Communication)

PROGRAM CONTACT:
PRISCILLA Novak
301-496-3136
priscilla.novak@nih.gov

Release Date: **03/03/2022**
Revised Date:

Application Number: **1 R01 AG078281-01**

Principal Investigator

POWELL, KIMBERLY RYAN

Applicant Organization: UNIVERSITY OF MISSOURI-COLUMBIA

Review Group: **ICSC**
Interdisciplinary Clinical Care in Specialty Care Settings Study Section

Meeting Date: **02/17/2022**
Council: **MAY 2022**
Requested Start: **07/01/2022**
RFA/PA: **PA20-185**
PCC: **2CHSRPN**

Project Title: **Reducing Avoidable Nursing Home-to-Hospital Transfers of Residents with ADRD: An Analysis of Interdisciplinary Team Communication using Text Messages**
SRG Action: **Impact Score:39 Percentile:32**
Next Steps: **Visit https://grants.nih.gov/grants/next_steps.htm**
Human Subjects: 30-Human subjects involved - Certified, no SRG concerns
Animal Subjects: 10-No live vertebrate animals involved for competing appl.
Gender: 1A-Both genders, scientifically acceptable
Minority: 1A-Minorities and non-minorities, scientifically acceptable
Age: 3A-No children included, scientifically acceptable

Project Year	Direct Costs Requested	Estimated Total Cost
1	200,000	313,670
2	225,000	352,879
3	225,000	352,879
TOTAL	650,000	1,019,429

ADMINISTRATIVE BUDGET NOTE: The budget shown is the requested budget and has not been adjusted to reflect any recommendations made by reviewers. If an award is planned, the costs will be calculated by Institute grants management staff based on the recommendations outlined below in the COMMITTEE BUDGET RECOMMENDATIONS section.
EARLY STAGE INVESTIGATOR
NEW INVESTIGATOR

1R01AG078281-01 Powell, Kimberly

EARLY STAGE INVESTIGATOR
NEW INVESTIGATOR

RESUME AND SUMMARY OF DISCUSSION: This application proposes to examine how interdisciplinary communication using text messaging (TM) can improve delivery of care to nursing home (NH) residents with Alzheimer's disease or a related dementia (ADRD), and prevent avoidable hospital transfer. The significance of the study lies in its ability to develop an intervention to improve team communication around NH to hospital transfers in order to reduce morbidity and mortality in NH residents with ADRD. The rigor of the prior research is grounded in the scientific literature and provides a solid foundation for the proposed study. The panel agreed that this early-stage principal investigator has relevant expertise and has assembled a highly successful investigative team that is compatible and would ensure the success of the project. The environment and institutional support is seen as excellent. The use of the 4M framework to analyze communication patterns is seen as a strength, however, some reviewers questioned that the application does not present details on why the 4M model will make a difference. The use of the MOQL dataset is also seen as a strength. During the discussion, the panel also noted some score-driving weaknesses, including the insufficient information about the use of TM within the NH, a lack of clarity about how the results will be applied to a new intervention, and the limited generalizability of the study findings outside of the target nursing homes. Additionally, some reviewers questioned why the communication patterns are only being applied to the analysis comparing ADRD versus non-ADRD. While these weaknesses did not detract from the potential high impact of this application for a handful of panel members, it did for the rest of the panelists who deemed it moderately impactful in offering valuable insights to improve team communication around NH to hospital transfers.

DESCRIPTION (provided by applicant): Close to two-thirds of the over 1.7 million U.S nursing home (NH) residents have a cognitive impairment such as Alzheimer's disease or a related dementia (ADRD), and are at high risk of hospital transfer. Reducing avoidable hospitalizations for NH residents is a national priority due to the negative effects on resident health and high Medicare and Medicaid costs. NH-to-hospital transfers result in > $2.6 billion in expenditures annually, harm to residents from physical and emotional relocation stress, and is avoidable in as many as 60% of cases. Avoidable NH-to-hospital transfers include transfers for conditions that can be safely and effectively managed in the NH. Early illness recognition and treatment could prevent the need for hospital transfer altogether, ultimately reducing morbidity and mortality in residents with ADRD while controlling costs. NH-to-hospital transfer decision-making is complex and relies on timely transmission of information among the interdisciplinary team. Unfortunately, many NHs rely on antiquated communication methods (e.g., phone, fax) rather than leveraging modern, convenient, and low-cost options, like text messaging (TM), which could improve health information sharing. Complicating the decision-making process about residents with ADRD is the progressive loss of language impacting the individual's ability to communicate. We expect that communication among health care team members differs for NH residents with and without ADRD. Our dataset provides an ideal and novel opportunity to apply natural language processing and social network analysis to TMs shared among an interdisciplinary team about NH resident transfer. We propose to examine the content of TM using the age-friendly health system 4M framework, which includes four evidence-based elements of high-quality care (what Matters, Medications, Mentation, and Mobility). The 4M framework addresses the core issues that should drive all decision making in the care of older adults and is a way of systematically rethinking care in ways that improve patient health and satisfaction. A critical need exists to examine how convenient, low-cost communication options like TM can reduce avoidable NH-to-hospital transfers of residents with ADRD. Our aims are to: 1) Identify documentation of the 4Ms in health information shared by the interdisciplinary team through electronic TM two weeks prior to NH-to-hospital transfer of residents with

ADRD; 2) Estimate the effects of the 4Ms found in TMs on avoidable NH-to-hospital transfers; and 3) Compare communication patterns of interdisciplinary teams making transfer decisions about residents with and without ADRD. A potential impact of this work is to decrease avoidable hospitalizations, and ultimately, morbidity and mortality in NH residents with ADRD by identifying evidence-based elements of high-quality care for older adults (4Ms). Another potential impact is the development of a structured language allowing for timely and seamless portability of information across the complete spectrum of care, optimizing the health of individuals and populations.

PUBLIC HEALTH RELEVANCE: In this project we examine how interdisciplinary communication using text messaging can improve delivery of age-friendly care to nursing home (NH) residents and prevent avoidable NH-to-hospital transfer. This study will provide information needed for the development and testing of interventions using convenient, low-cost technologies, like text messaging, to ultimately reduce morbidity and mortality in residents with Alzheimer's disease or a related dementia.

CRITIQUE 1

Significance: 3
Investigator(s): 2
Innovation: 3
Approach: 3
Environment: 2

Overall Impact: This study using a retrospective design will examine how interdisciplinary communication using text messaging (TM) can improve delivery of care to nursing home (NH) residents and prevent avoidable hospitalization. The investigators will identify documentation of the 4Ms (Matters, Medications, Mentation, and Mobility) in health information, estimate the effects of the 4Ms in TMs on avoidable hospitalizations, and compare communication patterns of teams making transfer decisions about NH residents with and without Alzheimer's Disease or a related dementia (ADRD). Pilot data revealed the most common messages sent and received were about patient updates (vital signs, lab results), confirmation of information , and questions about residents. Study team conducted 3 preliminary studies that revealed 1) timely communication will reduce NH to hospital transfers, 2) unknown how TM was used by different members of team, and 3) there were differences in low and high hospital transfer among NH residents (low transfer rates had more decision-making interactions). Inclusion across the lifespan has adequately been addressed. Sex as a biological variable has been discussed. The timeline is appropriate. The strengths of this proposal include: 1) preliminary data from investigative team, 2) examination of communication to reduce hospitalization, 3) investigators have collaborated previously. The weaknesses of this proposal include: 1) Limited to the previous data set (MOQI study), 2) Unclear how common for NHs to have access to TM. Considering the strengths and weaknesses of this proposal, the overall impact is moderate.

1. Significance:

Strengths

- 2/3 of 1.7 million US nursing home (NH) residents have cognitive impairment (e.g. Alzheimer's disease or a related dementia [ADRD]) at are at high risk for hospitalization.
- Early illness recognition and treatment could prevent hospitalization and reducing morbidity/mortality in residents with ADRD.
- NHs use antiquated communication methods to share health information.

- Potential of the development of a structure language that allows timely information across the spectrum of care.

Weaknesses

- Unclear how common for NHs to have access to TM

2. Investigator(s):

Strengths

- PI is a new and early stage nurse researcher and has Senior researcher/mentor on study
- Several investigators on team have worked on prior studies and published together.
- Investigator with expertise in computer engineering is on the team.
- A biostatistician is on the team.
- Only one study focusing on TM communication included Nurses

Weaknesses

- None noted by reviewer.

3. Innovation:

Strengths

- Use of the 4M framework to analyze communication patterns.
- The examination of TM communication between providers in a field where phone/fax are the status quo.

Weaknesses

- TM has been examined before, but in a different discipline.

4. Approach:

Strengths

- The proposal is guided by the 4M framework.
- Thorough development of 4M taxonomy
- Use of the MOQI dataset (investigators have ready access)
- Plan to triangulate findings by using multiple data sources and methods

Weaknesses

- Limited to the previous data set (MOQI study)

5. Environment:

Strengths

- University of Missouri and Columbia University have the capabilities and support to successfully complete this proposal.

Weaknesses

- None noted by reviewer.

Protections for Human Subjects

Not Applicable (No Human Subjects)

Data and Safety Monitoring Plan (Applicable for Clinical Trials Only):

Not Applicable (No Clinical Trials)

Inclusion Plans

- Sex/Gender: Distribution justified scientifically
- Race/Ethnicity: Distribution justified scientifically
- For NIH-Defined Phase III trials, Plans for valid design and analysis: Not applicable
- Inclusion/Exclusion Based on Age: Distribution justified scientifically

Vertebrate Animals

Not Applicable (No Vertebrate Animals)

Biohazards

Not Applicable (No Biohazards)

Budget and Period of Support

Recommend as Requested

CRITIQUE 2

Significance: 2
Investigator(s): 1
Innovation: 2
Approach: 4
Environment: 1

Overall Impact: The goal of this study is to use NLP and social network analysis to study text-messages prior to hospital transfers for nursing home patients. The team proposes use standardized vocabularies, clinical expertise and NLP to document 4Ms in text-message communications (Aim 1), examine the associations between the 4Ms and transfers (Aim 2), compare communications prior to transfers for ADRD patients vs. non ADRD patients (Aim 3). This study leverages text messages from a multi-years MOQI study funded by AHRQ. Strengths of this study include a strong investigative team, rich text-message data set, focus on a significant and potentially modifiable aspect of healthcare costs for NH, strong preliminary data. The main limitation is potential for lack of generalizability due to poor use of text messages and no descriptive information about the NH included in this study (how representative). In addition, it is not clear why the communication patterns are only being applied to the analysis comparing ADRD vs. non ADRD. Finally, it would be helpful to have a greater understanding of how results of this study could be applied to new interventions. In summary, this application has the

potential to advance interventions to improve team communication around NH to hospital transfers and has only a few minor limitations.

1. Significance:

Strengths

- This project focuses on a costly and potentially modifiable healthcare problem.
- The authors identify that improving communication among the team has the potential to improve outcomes, patient quality of life and reduce costs.
- The research is founded on a strong scientific premise and supported by a strong literature of team based care in other settings.

Weaknesses

- The concept of avoidable transfers is not entirely clear in this project (appears that it is rated by APRNs after the event).
- Communication is only one aspect of care, other things such as staffing ratio, SES or other factors may also play a role.
- It would be helpful to have more information about how this data could be use in an intervention, especially considering the NH in this study appear to be already participating in an intervention.

2. Investigator(s):

Strengths

- Dr. Powell is an early-stage investigator and well-suited to be the PI for this project
- Co-investigators Popescu, Mehr, Lee and Alexander provide appropriate expertise to support the project.

Weaknesses

- None noted by reviewer.

3. Innovation:

Strengths

- Application of NLP and social network analysis to NH transfers is novel.

Weaknesses

- Results of this study are an incremental step toward improving team communication to prevent NH transfers

4. Approach:

Strengths

- Rich existing dataset available for this study.
- Preliminary data analysis from only 6 months demonstrates the team’s expertise and also support the proposed relationships.

Weaknesses

- Possible lack of generalizability of the NH enrolled in the prior study. It appears they were participating in an intervention. In addition, the text message may lack generalizability because as the authors point out, NH teams are often not communicating by text.
- Not clear why the team's interaction is only being compared between the ADRD and non ADRD samples, why not compare also between the preventable and non-preventable transfers?

5. Environment:

Strengths

- Appropriate for completing the proposed study

Weaknesses

- None noted by reviewer.

Protections for Human Subjects

Acceptable Risks and/or Adequate Protections

Data and Safety Monitoring Plan (Applicable for Clinical Trials Only):

Acceptable

Inclusion Plans

- Sex/Gender: Distribution justified scientifically
- Race/Ethnicity: Distribution justified scientifically
- For NIH-Defined Phase III trials, Plans for valid design and analysis: Not applicable
- Inclusion/Exclusion Based on Age: Distribution justified scientifically

Vertebrate Animals

Not Applicable (No Vertebrate Animals)

Biohazards

Not Applicable (No Biohazards)

Budget and Period of Support

Recommend as Requested

CRITIQUE 3

Significance: 2
Investigator(s): 3
Innovation: 3
Approach: 3
Environment: 1

Overall Impact: The majority of nursing home residents in the U.S. have a cognitive impairment and are at high risk of hospital transfer, and reducing hospital transfer is important due to health and financial reasons. This proposed project plans to investigate how interdisciplinary communication using text messaging can improve care delivery to nursing home residents with Alzheimer's disease or a related dementia (ADRD), and prevent avoidable hospital transfer. The study team offers adequate expertise and experience for successfully carrying out the proposed research. The environment and institutional support is excellent. Research design, methods and timeline are well presented. One concern is that the PI may need to demonstrate her independent role in leading such an significant project due to her limited experience in working with ADRD patients.

1. Significance:

Strengths

- Two-thirds of nursing home residents in the U.S. have a cognitive impairment and are at high risk of hospital transfer.
- Reducing hospitalizations for nursing home residents is important due to health and financial concerns.
- This proposed project will examine how interdisciplinary communication using text messaging can improve care delivery to nursing home residents with Alzheimer's disease or a related dementia (ADRD), and prevent avoidable nursing home-to-hospital transfer.

Weaknesses

- None noted by reviewer.

2. Investigator(s):

Strengths

- PI is a new and early-stage nurse researcher with an interest in health information sharing.
- PI has served as postdoc or co-I on grants, and has a good track record of publications.
- Co-Is have commentary areas of expertise and are well suit for this project.

Weaknesses

- PI's work mostly overlaps with the postdoc mentor
- PI has a limited experience in studying the ADRD population.

3. Innovation:

Strengths

- This study will build upon previous work by using an innovative conceptual framework to examine the content of text messages and leveraging advanced methods.

Weaknesses

- None noted by reviewer.

4. Approach:

Strengths

- Approach is well presented with a clear plan.
- Data collection and analysis is well presented.

Weaknesses

- Scenically relevant preliminary work is presented, although is placed in the significance section.

5. Environment:

Strengths

- Institutional support and commitment is strong.

Weaknesses

- None noted by reviewer.

Protections for Human Subjects

Acceptable Risks and/or Adequate Protections

- In place

Data and Safety Monitoring Plan (Applicable for Clinical Trials Only):

Not Applicable (No Clinical Trials)

Inclusion Plans

- Sex/Gender: Distribution justified scientifically
- Race/Ethnicity: Distribution justified scientifically
- For NIH-Defined Phase III trials, Plans for valid design and analysis: Not applicable
- Inclusion/Exclusion Based on Age: Distribution justified scientifically
- 36% Male, 64% Female; 75% White, 22% Black, 2% Asian, 1% More than One Race; 54-102 Years of Age

Vertebrate Animals

Not Applicable (No Vertebrate Animals)

Biohazards

Not Applicable (No Biohazards)

Resource Sharing Plans

Unacceptable

Budget and Period of Support

Recommend as Requested

THE FOLLOWING SECTIONS WERE PREPARED BY THE SCIENTIFIC REVIEW OFFICER TO SUMMARIZE THE OUTCOME OF DISCUSSIONS OF THE REVIEW COMMITTEE, OR REVIEWERS' WRITTEN CRITIQUES, ON THE FOLLOWING ISSUES:

PROTECTION OF HUMAN SUBJECTS: ACCEPTABLE

INCLUSION OF WOMEN PLAN: ACCEPTABLE

INCLUSION OF MINORITIES PLAN: ACCEPTABLE

INCLUSION ACROSS THE LIFESPAN: ACCEPTABLE

COMMITTEE BUDGET RECOMMENDATIONS: The budget was recommended as requested.

Footnotes for 1 R01 AG078281-01; PI Name: Powell, Kimberly Ryan

NIH has modified its policy regarding the receipt of resubmissions (amended applications).See Guide Notice NOT-OD-18-197 at https://grants.nih.gov/grants/guide/notice-files/NOT-OD-18-197.html. The impact/priority score is calculated after discussion of an application by averaging the overall scores (1-9) given by all voting reviewers on the committee and multiplying by 10. The criterion scores are submitted prior to the meeting by the individual reviewers assigned to an application, and are not discussed specifically at the review meeting or calculated into the overall impact score. Some applications also receive a percentile ranking. For details on the review process, see http://grants.nih.gov/grants/peer_review_process.htm#scoring.

MEETING ROSTER

Interdisciplinary Clinical Care in Specialty Care Settings Study Section
Healthcare Delivery and Methodologies Integrated Review Group
CENTER FOR SCIENTIFIC REVIEW
ICSC
02/17/2022 - 02/18/2022

Notice of NIH Policy to All Applicants: Meeting rosters are provided for information purposes only. Applicant investigators and institutional officials must not communicate directly with study section members about an application before or after the review. Failure to observe this policy will create a serious breach of integrity in the peer review process, and may lead to actions outlined in NOT-OD-14-073 at https://grants.nih.gov/grants/guide/notice-files/NOT-OD-14-073.html, NOT-OD-15-106 at https://grants.nih.gov/grants/guide/notice-files/NOT-OD-15-106.html, and NOT-OD-18-115 at https://grants.nih.gov/grants/guide/notice-files/NOT-OD-18-115.html, including removal of the application from immediate review.

<u>**CHAIRPERSON(S)**</u>

COMPTON, PEGGY A, PHD
PROFESSOR
DEPARTMENT OF FAMILY AND COMMUNITY HEALTH
SCHOOL OF NURSING
UNIVERSITY OF PENNSYLVANIA
PHILADELPHIA, PA 19104

<u>**MEMBERS**</u>

BAILEY, STACY C, PHD *
ASSOCIATE PROFESSOR
DIVISION OF GENERAL INTERNAL MEDICINE & GERIATRICS
FEINBERG SCHOOL OF MEDICINE
NORTHWESTERN UNIVERSITY
CHICAGO, IL 60611

BARON, KELLY GLAZER, PHD *
ASSOCIATE PROFESSOR
DIVISION OF PUBLIC HEALTH
DEPARTMENT OF FAMILY AND PREVENTIVE MEDICINE
UNIVERSITY OF UTAH
SALT LAKE CITY, UT 84108

BELL, ALEECA F, PHD *
ASSOCIATE PROFESSOR
COLLEGE OF NURSING
UNIVERSITY OF ARIZONA
TUCSON, AZ 85721

BLAKE, KATHRYN V, PHARMD
DIRECTOR, CENTER FOR PHARMACOGENOMICS AND TRANSLATIONAL RESEARCH
RESEARCH DEPARTMENT
NEMOURS CHILDREN'S HEALTH
JACKSONVILLE, FL 32207

BRIENZA, DAVID MICHAEL, PHD
PROFESSOR
DEPARTMENT OF REHAB SCI & TECHNOLOGY
UNIVERSITY OF PITTSBURGH
PITTSBURGH, PA 15260

CHAO, CHUN R, PHD *
RESEARCH SCIENTIST
DEPARTMENT OF RESEARCH AND EVALUATION
KAISER PERMANENTE SOUTHERN CALIFORNIA
PASADENA, CA 91101

CHAUDHARI, AJIT MOHAN WORTHEN, PHD *
PROFESSOR AND DIRECTOR OF DIVERSITY, EQUITY, AND INCLUSION
SCHOOL OF HEALTH AND REHABILITATION SCIENCES
THE OHIO STATE UNIVERSITY
COLUMBUS, OH 43210

CHEN, RONALD, MD
PROFESSOR AND CHAIR
DEPARTMENT OF RADIATION ONCOLOGY
UNIVERSITY OF KANSAS MEDICAL CENTER
KANSAS CITY, KS 66160

CHU, DAVID, MD *
ASSISTANT PROFESSOR
DIVISION OF UROLOGY
LURIE CHILDREN'S HOSPITAL OF CHICAGO
CHICAGO, IL 60611-2991

DAIL, ROBIN BRITT, PHD
PROFESSOR AND ASSOCIATE DEAN FOR FACULTY AFFAIRS
COLLEGE OF NURSING
UNIVERSITY OF SOUTH CAROLINA
COLUMBIA, SC 29208

DOORENBOS, ARDITH Z, PHD *
PROFESSOR
BIOBEHAVIORAL NURSING SCIENCE
COLLEGE OF NURSING
UNIVERSITY OF ILLINOIS, CHICAGO
CHICAGO, IL 60612

DRAWZ, PAUL ENGLUND, MD *
ASSOCIATE PROFESSOR OF MEDICINE
DEPARTMENT OF MEDICINE
DIVISION OF RENAL DISEASES AND HYPERTENSION
UNIVERSITY OF MINNESOTA
MINNEAPOLIS, MN 55414

DRENDEL, AMY L, DO *
PROFESSOR OF PEDIATRICS
CHILDREN'S WISCONSIN
MEDICAL COLLEGE OF WISCONSIN
MILWAUKEE, WI 53201

FERRANTE, LAUREN, MD *
ASSISTANT PROFESSOR OF MEDICINE
SECTION OF PULMONARY, CRITICAL CARE,
AND SLEEP MEDICINE
DEPARTMENT OF INTERNAL MEDICINE
YALE SCHOOL OF MEDICINE
NEW HAVEN, CT 06520

FISCHER, STACY M, MD
ASSOCIATE PROFESSOR
DEPARTMENT OF MEDICINE
DIVISION OF GENERAL INTERNAL MEDICINE
UNIVERSITY OF COLORADO
AURORA, CO 80045

FRIDMAN, ESTEBAN ANDRES, MD, PHD *
ASSISTANT PROFESSOR OF NEUROSCIENCE
DIVISION OF SYSTEM NEUROLOGY AND NEUROSCIENCE
BRAIN AND MIND RESEARCH INSTITUTE
WEILL CORNELL MEDICAL COLLEGE
CORNELL UNIVERSITY
NEW YORK, NY 10065

GEORGE, MAUREEN, PHD *
PROFESSOR
SCHOOL OF NURSING
COLUMBIA UNIVERSITY
NEW YORK CITY, NY 10032

GROTH, SUSAN W, PHD
ASSOCIATE PROFESSOR
SCHOOL OF NURSING
UNIVERSITY OF ROCHESTER
ROCHESTER, NY 14642

HICKMAN, RONALD LEE JR, PHD
ASSOCIATE PROFESSOR AND ASSOCIATE DEAN OF
RESEARCH
FRANCES PAYNE BOLTON SCHOOL OF NURSING
CASE WESTERN RESERVE UNIVERSITY
CLEVELAND, OH 44118

HUBBARD, REBECCA, PHD
PROFESSOR
DEPARTMENT OF BIOSTATISTICS, EPIDEMIOLOGY
AND INFORMATICS
UNIVERSITY OF PENNSYLVANIA
PHILADELPHIA, PA 19104

HUI, DAVID, MD
ASSOCIATE PROFESSOR
DEPARTMENT OF PALLIATIVE CARE,
REHABILITATION AND INTEGRATIVE MEDICINE
DEPARTMENT OF GENERAL ONCOLOGY
UNIVERSITY OF TEXAS MD ANDERSON CANCER CENTER
HOUSTON, TX 77030

HUR, CHIN, MD
PROFESSOR OF MEDICINE AND EPIDEMIOLOGY
COLUMBIA UNIVERSITY MEDICAL CENTER
NEW YORK, NY 10032

JATOI, AMINAH, MD
BETTY J. FOUST, MD AND PARENTS' PROFESSOR OF
ONCOLOGY
DEPARTMENT OF ONCOLOGY
MAYO CLINIC
ROCHESTER, MN 55905

JONES, RANDY ALLEN, PHD
PROFESSOR AND ASSOCIATE DEAN FOR PARTNER
DEVELOPMENT AND ENGAGEMENT
ASSISTANT DIRECTOR, COMMUNITY OUTREACH
AND ENGAGEMENT, CANCER CENTER
SCHOOL OF NURSING
UNIVERSITY OF VIRGINIA
CHARLOTTESVILLE, VA 22904-4195

LAPANE, KATE L, PHD
ASSOCIATE DEAN, PROFESSOR AND CHIEF
DEPARTMENT OF QUANTITATIVE HEALTH SCIENCES
DIVISION OF EPIDEMIOLOGY
UNIVERSITY OF MASSACHUSETTS
WORCESTER, MA 01655

LARSON, JANET, PHD
PROFESSOR AND CHAIR
HEALTH BEHAVIOR AND BIOLOGICAL SCIENCES
SCHOOL OF NURSING
UNIVERSITY OF MICHIGAN
ANN ARBOR, MI 48109

LYON, DEBRA E, PHD
PROFESSOR AND EXECUTIVE ASSOCIATE DEAN
DEPARTMENT OF BIOBEHAVIORAL NURSING SCIENCES
COLLEGE OF NURSING
UNIVERSITY OF FLORIDA
GAINESVILLE, FL 32610

LYON, MAUREEN ELLEN, PHD
PROFESSOR OF PEDIATRICS
CHILDREN'S NATIONAL HOSPITAL
SCHOOL OF MEDICINE AND HEALTH SCIENCES
GEORGE WASHINGTON UNIVERSITY
WASHINGTON, DC 20010

MACK, JENNIFER W, MD
ASSOCIATE PROFESSOR
DEPARTMENT OF PEDIATRICS
DANA-FARBER CANCER INSTITUTE
HARVARD MEDICAL SCHOOL
BOSTON, MA 02215

MAO, JUN J, MD
LAURANCE S. ROCKEFELLER CHAIR
PROFESSOR AND CHIEF, INTEGRATIVE MEDICINE SERVICE
DEPARTMENT OF MEDICINE
MEMORIAL SLOAN KETTERING CANCER CENTER
NEW YORK, NY 10021

MAUCK, MATTHEW CHRISTOPHER, MD, PHD *
ASSISTANT PROFESSOR
DEPARTMENT OF ANESTHESIOLOGY
SCHOOL OF MEDICINE
UNIV OF NORTH CAROLINA CHAPEL HILL
CHAPEL HILL, NC 27599

PINEDA, ROBERTA, PHD *
ASSISTANT PROFESSOR
CHAN DIVISION OF OCCUPATIONAL SCIENCE
AND OCCUPATIONAL THERAPY
DEPARTMENT OF PEDIATRICS
UNIVERSITY OF SOUTHERN CALIFORNIA
LOS ANGELES, CA 90089-9003

STULBERG, DEBRA, MD *
ASSOCIATE PROFESSOR AND CHAIR
DEPARTMENT OF FAMILY MEDICINE
UNIVERSITY OF CHICAGO
CHICAGO, IL 60637

YAP, TRACEY, PHD
ASSOCIATE PROFESSOR
SCHOOL OF NURSING
DUKE UNIVERSITY
DURHAM, NC 27710

YOU, TONGJIAN, PHD *
PROFESSOR AND CHAIR
DEPARTMENT OF EXERCISE AND HEALTH SCIENCES
ROBERT AND DONNA MANNING COLLEGE
OF NURSING AND HEALTH SCIENCES
UNIVERSITY OF MASSACHUSETTS BOSTON
BOSTON, MA 02125

SCIENTIFIC REVIEW OFFICER

ABDULLAH, ABU SALEH MOHAMMAD, PHD
SCIENTIFIC REVIEW OFFICER
CENTER FOR SCIENTIFIC REVIEW
NATIONAL INSTITUTES OF HEALTH
6701 ROCKLEDGE DRIVE
BETHESDA, MD 20892

EXTRAMURAL SUPPORT ASSISTANT

JONES, BELINDA
EXTRAMURAL SUPPORT ASSISTANT
CENTER FOR SCIENTIFIC REVIEW
NATIONAL INSTITUTES OF HEALTH
BETHESDA, MD 20892

* Temporary Member. For grant applications, temporary members may participate in the entire meeting or may review only selected applications as needed.

Consultants are required to absent themselves from the room during the review of any application if their presence would constitute or appear to constitute a conflict of interest.
